# Low prevalence of methotrexate intolerance in rheumatoid arthritis: a South African study

**DOI:** 10.1007/s10067-025-07310-5

**Published:** 2025-02-06

**Authors:** Namuhla Qwabe, Farhanah Paruk, Girish Mahasukhlal Mody

**Affiliations:** 1 Department of Rheumatology, School of Clinical Medicine, College of Health Science, University of Kwa-Zulu Natal, Durban, South Africa; 2 Division of Internal Medicine, University of KwaZulu Natal, Durban, South Africa

**Keywords:** Africa, African Blacks, Indians, Intolerance, Methotrexate, Rheumatoid arthritis

## Abstract

**Introduction:**

Methotrexate (MTX) is the first line therapy for rheumatoid arthritis (RA), and despite its widespread use, there is very little information about MTX intolerance in sub-Saharan Africa. The aim of this study was to assess the prevalence of MTX intolerance and other reasons for stopping MTX in RA in South Africa.

**Methods:**

A retrospective chart review of all RA patients seen at Inkosi Albert Luthuli Hospital in Durban from 2009 to 2019 was undertaken. We included patients who received MTX for at least three months. All patients received folic acid supplements. Patients who discontinued MTX were categorized as having either MTX related toxicity or other reasons.

**Results:**

A total of 695 patients were identified with a female to male ratio of 7:1. The mean age was 57.9 (± 13.3) years, and median duration of MTX use was 67.0 (39.0—106.0) months. Most of the patients were African Blacks (61.2%), and Indians (32.8%). There were 83 (11.9%) patients who stopped MTX, and it was successfully reintroduced in 25 of them. Thus, 58 (8.3%) patients discontinued therapy, 33 (4.7%) due to intolerance and 25 (3.6%) due to factors other than adverse effects. The commonest causes of MTX intolerance were respiratory, gastrointestinal and haematological. The other reasons for discontinuation included co-morbidities and pregnancy related concerns.

**Conclusions:**

The low prevalence of MTX intolerance in a multiethnic population in this single centre study, confirms the value of MTX as anchor therapy, especially in resource constrained settings.

**Table:** 

**Key Points** • *We report a low and similar prevalence of methotrexate intolerance in a large population of African Blacks and Indians with RA in sub-Saharan Africa.* • *Even though there was heterogeneity among other studies, our review indicates that MTX was tolerated better in our patients compared to patients in Europe and the United States of America.*

## Introduction

Methotrexate (MTX) is used in the management of rheumatoid arthritis (RA) since the 1980’s and is more effective in reducing inflammation, structural damage and disability than other synthetic disease modifying anti-rheumatic drugs (DMARD) [1]. Methotrexate is widely available, has an acceptable safety profile, is cost effective and is associated with reduced cardiovascular mortality [2, 3]. Current treatment guidelines recommend MTX as initial monotherapy in DMARD naïve patients, and it is also used in combination with other synthetic DMARDs and biological agents [4, 5]. These guidelines and other systematic reviews include recommendations for baseline screening prior to the initiation of MTX and monitoring for adverse effects, but there is no uniformity, and modifications are necessary, based on local practice and disease profiles [6].

A systematic review of 88 studies of RA patients treated with MTX monotherapy showed that the discontinuation rates due to intolerance or toxicity ranged from 10—37% [2]. Analysis of 3463 RA patients in 21 prospective cohorts who received therapy for at least two years, found that even though 72.9% of the patients developed an adverse event, there was permanent discontinuation due to toxicity in only 10.5% of patients [2]. The most common adverse effects reported with MTX therapy are gastrointestinal, hepatic, haematological, pulmonary, infections and cutaneous.

A 2014 Cochrane database review showed a discontinuation rate of 16% in short term (12—52 week) studies of MTX monotherapy in RA [7]. A large single centre United Kingdom (UK) study of 1257 patients on MTX, noted a withdrawal rate of 34% (260 of 762) among RA patients and 37% (71 of 193) in patients with psoriatic arthritis [8]. Main reasons for discontinuation of therapy in the RA patients were adverse effects in 76.9% (gastrointestinal, respiratory, haematological, neurological), followed by lack of efficacy in 12.3% and other factors 10.8%.

There is a paucity of data regarding the tolerance of MTX among African Blacks with RA in sub-Saharan Africa (SSA). Hitchon et al. have drawn attention to the challenges faced by doctors with respect to the use of MTX in Africa [9]. Zongo et al. reported a series of 205 RA patients in Burkina Faso and 28.8% of their 135 MTX treated patients experienced an adverse effect. However, discontinuation of MTX was due to an adverse effect in 5.9%, while shortage of medication, pregnancy related concerns, and financial and social factors accounted for discontinuation in 21.4% [10].

A recent meta-analysis showed a relationship between ATIC 347 C/G polymorphism and non-responsiveness to MTX in Europeans but not in Asians, however, there was no correlation with toxicity [11]. Therefore, information about the responsiveness or toxicity is important in the search for genetic factors in all ethnic groups.

In view of limited data in SSA on the reasons for discontinuation of MTX, this study was undertaken to determine the prevalence of drug intolerance and other reasons for stopping MTX and to determine whether there are any ethnic differences in RA patients seen in a rheumatology department at a university affiliated hospital in South Africa (SA).

## Patients and methods

We conducted a retrospective review of the electronic hospital records of all the patients classified as having RA who were seen in the Department of Rheumatology at Inkosi Albert Luthuli Central Hospital (IALCH) in Durban, SA from January 2009 to December 2019.

Patients who fulfilled the 1987 American College of Rheumatology or 2010 American College of Rheumatology /European League Against Rheumatism classification criteria [12, 13], were MTX naïve, had baseline laboratory data available and received MTX for at least three months, were included in the study.

Rheumatoid arthritis patients who were known or tested positive for human immunodeficiency virus (HIV), had features of another connective tissue disease such as systemic lupus erythematous, scleroderma, idiopathic inflammatory myopathies, or mixed connective tissue disease, had prior treatment with MTX, and if they had received MTX for less than three months were excluded.

The data recorded included the demographic data (age, gender, ethnicity, and employment status), presence of co-morbidities, duration of RA, and laboratory results (full blood count, liver function tests, urea and creatinine, rheumatoid factor (RF), anti-cyclic citrullinated peptide antibody (ACPA), erythrocyte sedimentation rate (ESR) and C- reactive protein (CRP.) The initial and most recent dose of MTX, duration of MTX use, and reasons for discontinuation were documented. The reasons for discontinuation of MTX were classified as being due to drug toxicity or factors other than toxicity, which included social factors. Prior to initiation of MTX, all patients were tested for hepatitis B and C virus, HIV and had a baseline chest radiograph.

The standard approach to RA management in our department was to commence methotrexate therapy 10 mg per week and folic acid 5 mg on five days a week unless contraindicated. The dose of MTX was escalated to a maximum of 25 mg per week if the response was inadequate. If the disease activity was still not controlled, or a flare occurred after an initial response, then chloroquine was added, followed by salazopyrine if necessary. Patients who still had active disease were then treated with leflunomide in combination with MTX, and chloroquine and salazopyrine were stopped. Disease activity scores were not available for all patients and therefore the initial and latest ESR and CRP values were used to assess response to MTX therapy.

Ethical approval for the study was granted by the Biomedical Research Ethics Committee of University of KwaZulu-Natal (BREC number 00003105/21), and approval for the study was obtained from the KwaZulu-Natal Provincial Department of Health and IALCH.

## Statistical methods

Descriptive statistics are reported for demographic data**,** clinical characteristics, and MTX intolerance. Categorical variables are summarized using frequencies (with percentages), and continuous variables are summarized using the mean and standard deviation (SD) for normally distributed continuous data, or the median, with lower and upper quartiles (IQR, inter-quartile range) for skewed continuous data and count (percentages). We used Pearson’s Chi Squared test or Fisher’s exact test for comparison of means and medians to assess the association between categorical variables between the Black African and Indian ethnic groups. Reasons for discontinuation and toxicity were grouped according to the system involved (haematological, gastrointestinal etc.) and reported as total and per African and Indian population group. The level of significance was set at p < 0.05. All statistical analyses were performed using SPSS® version 24 (IBM Corp. Released 2023. IBM SPSS Statistics for Windows, Version 29.0.2.0 Armonk, NY: IBM Corp).

## Results

### Demographic data, comorbidities, laboratory findings and treatment in the different ethnic groups

Of the 695 patients, the majority 596 (85.8%) of the patients fulfilled the 2010 ACR criteria and 608 (87.5%) were females with a female to male ratio of 7:1. The mean age was 57.9 (± 13.3) years. Most of the patients were African Blacks (61.2%) followed by Indians (32.8%), Whites (3.9%) and Mixed Ethnicity (2.2%) as shown in Table 1. The median duration of RA was 72.0 (38.0—101.0) months and the median duration of MTX therapy was 67.0 (39.0—106.0) months and the mean initial dose of MTX was 9.9 ± 0.1 mg/week. The median MTX duration was 68.5 (40.0—108.7) months in females and was significantly greater (p = 0.014) than 60.0 (30.0—84.0) months for males.

**Table 1:** Comparison of the demographic data, comorbidities, laboratory findings and treatment in the different ethnic groups with rheumatoid arthritis

	African (*n* = 425) n(%)	Indian(*n* = 228) n(%)	White (*n* = 27) n(%)	Mixed ethnicity (*n* = 15) n(%)	Total (*n* = 695) n(%)	Comparison of African and Indians (p-value)
Age in years (mean) ± SD	57.2 ± 13.8	59.1 ± 12.3	58.7 ± 12.1	55.6 ± 12.4	57.9 ± 13.3	0.102
Females	379 (89.2)	196 (86.0)	20 (74.1)	13 (86.7)	608 (87.5)	0.228
RA duration (months) median (IQR)	68.0 (32–98.5)	79.0 (44.0–111.5)	76.0 (44.0–87.0)	74.0 (27.0–110.0)	72.0 (38.0–101.0)	< 0.001
Duration of MTX (months) median (IQR)	62.0 (33.0–103.0)	78.0 (50.2–105.0)	65.0 (29.0–98.0)	73.0 (30.0–86.0)	67.0 (39.0–106.0)	0.005
Habits: Smoker (ever) *n* = 689	36 (8.5)	61 (26.8)	14 (51.9)	8(53.3)	119 (17.3)	< 0.001
Alcohol (ever) *n* = 688	27 (6.4)	23 (10.1)	5 (18.5)	2 (13.3)	57 (8.3)	0.100
Comorbidities						
Hypertension	211 (49.6)	124 (54.4)	11 (40.7)	8 (53.3)	354 (50.9)	0.249
Diabetes	59 (13.9)	65 (28.5)	2 (7.4)	5 (33.3)	131 (18.8)	< 0.001
Ischaemic heart disease	8 (1.9)	17 (7.5)	3 (11.1)	1 (6.7)	29 (4.2)	0.010
Renal disease	33 (7.8)	31 (13.6)	1 (3.7)	1 (6.7)	66 (9.5)	0.008
Liver disease	1 (0.2)	2 (0.9)	0 (0.0)	0 (0.0)	3 (0.4)	0.248
Positive serology						
Rheumatoid factor (RF)	364 (85.6)	198 (86.8)	26 (96.3)	14 (93.3)	602 (86.6)	0.470
ACPA	346 (81.4)	200 (87.7)	24 (88.9)	14 (93.3)	584 (84.0)	0.744
RF or ACPA	400 (94.1)	220 (96.5)	26 (96.3)	15 (100)	661 (95.1)	0.292
Inflammatory markers (median; IQR)						
Initial CRP (mg/dL)	22.0 (10.0–46.5)	15.0 (8.0–35.7)	35.0 (13.0–53.0)	24.0 (6.0–39.0)	20.0 (9.0–43.0)	0.002
Last visit CRP (mg/dL)	13.0 (5.0–26.0)	10.0 (4.0–22.7)	14.0 (4.0–24.0)	16.0 (4.0–32.0)	12.0 (4.0–25.0)	0.024
CRP change over time (p-value)	< 0.001	< 0.001	< 0.001	0.601	< 0.001	
Initial ESR (mm/hour)	53.0 (32.0–79.0)	38.0 (26.2–62.0)	39.0 (17.0–55.0)	38.0 (28.0–46.0)	45.0 (28.0–73.0)	< 0.001
Last visit ESR (mm/ hour)	37.0 (23.0–56.5)	33.5 (19.0–51.0)	28.0 (11.0–45.0)	25.0 (17.0–38.0)	35.0 (20.0–53.0)	0.071
ESR change over time p value	< 0.001	< 0.001	0.036	0.219	< 0.001	
Medication						
Salazopyrine	156 (36.7)	79 (34.6)	13 (48.1)	5 (33.3)	253 (36.4)	0.455
Chloroquine	344 (80.9)	173 (75.9)	20 (74.1)	8 (53.3)	545 (78.4)	0.074
Leflunomide	31 (7.3)	21 (9.2)	2 (7.4)	1 (6.7)	55 (7.9)	0.331
Prednisone use	373 (87.8)	194 (85.1)	26 (96.3)	15 (100)	608 (87.5)	0.254
Prednisone dose (mean) ± SD	5.06 ± 0.79	5.13 ± 1.07	5.0 ± 0.0	5.0 ± 0.0	5.08 ± 0.90	0.305

*RA* rheumatoid arthritis, *MTX* methotrexate, *ACPA* anticitrullinated peptide antibody, *ESR* erythrocyte sedimentation ratio, *CRP* C-reactive protein

-Of the total, 56% of the patients were unemployed, 27% were pensioners and only 17% were employed. A history of ever smoking cigarettes was reported in 17.3% and alcohol intake in 8.3%. The most common co-morbidities were hypertension (50.9%) followed by diabetes (18.8%). There were three (0.4%) patients who had stable liver disease (one had history of gallstones, and two had non-alcoholic fatty liver disease).

Majority of the patients had a positive RF test (86.6%) and ACPA (84.0%) with 95.1% being positive for either one of the tests. There was a statistically significant improvement (p < 0.001) in both the ESR in mm/hour and the C-reactive protein (mg/dl) from the initial visit to the last visit as shown in Table 1. Prednisone was used in low doses in 87.5% of patients, and the other DMARDS prescribed were chloroquine (78.4%), salazopyrine (36.4%), and leflunomide (7.9%).

### Comparison of the findings between African Black and Indian patients

Due to the small number of Whites and patients of Mixed Ethnicity, ethnic comparison was undertaken only between the African Blacks and Indians (Table 1). There was no difference in the age, gender, and occupational status between the groups. There was a higher prevalence of smoking (p < 0.001), diabetes (p < 0.001), ischaemic heart disease (p = 0.010) and renal disease (p = 0.008) among Indian patients. The African Blacks had significantly higher levels of ESR and CRP at the initial and last visits when compared to Indian patients, and both groups showed a significant improvement after treatment with MTX (Table 1). There was no difference in the use of prednisone and other synthetic DMARDS between the groups, but Indian patients used MTX for a significantly longer median duration of 78 (50.2—105.0) months compared 62 (33.0—103.0) months; (p = 0.003) for African Blacks.

### Reasons for methotrexate discontinuation

Table 2 shows that 83 (11.9%) of the patients discontinued MTX therapy, of whom 44 (6.3%) were initially attributed to MTX toxicity, and 39 (5.6%) were unrelated to MTX use. The reasons for discontinuation due to MTX toxicity were interstitial lung disease (ILD) in 15 (2.2%) patients, gastrointestinal intolerance in 12 (1.7%; severe nausea alone in five, nausea and vomiting in three and nausea and abdominal pain in four), bone marrow toxicity in seven (1.0%; leucopaenia in five and thrombocytopaenia in two patients), infection in eight (1.2%; tuberculosis in three, bacterial infection in three and one each had viral and fungal infection).

**Table 2:** Reasons for the discontinuation of methotrexate and successful rechallenge

	Africans (*n* = 425) n (%)	Indians (*n* = 228) n (%)	Whites (*n* = 27) n (%)	^#^Total (*n* = 695) n (%)	Successful Rechallenge n (%)	Total who stopped MTX *n* = 695 n (%)
MTX discontinuation related to toxicity	**24 (5.6%)**	**20 (8.8%)**	**0**	**44 (6.3%)**	**11 (25%)**	**33 (4.7%)**
Bone marrow toxicity	5	2	0	7	2	5 (.0.9)
Leucopaenia	4	1	0	5	2	3 (0.4)
Thrombocytopaenia	1	1	0	2	0	2 (0.3)
GI intolerance	6	6	0	12	3	9 (1.3)
Interstitial lung disease	6	9	0	15	1	14(2.0)
Hepatotoxicity	1	1	0	2	1	1(0.1%)
Infection	6	2	0	8	4	4 (0.6)
MTX discontinuation unrelated to toxicity	**28 (6.6%)**	**9 (4.0%)**	**2 (7.4%)**	**39 (5.6%)**	**14 (35.8)**	**25 (3.6%)**
Iron deficiency anaemia	16	7	1	24	10	14 (2.0)
Declining renal function	1	1	0	2	0	2 (0.3)
Desiring conception	6	1	0	7	2	5 (0.7
Pregnancy	3	0	1	4	2	2 (0.3)
Malignancy	2	0	0	2	0	2 (0.3)

*MTX* methotrexate, *GI* gastrointestinal

^#^There were no discontinuations in the Mixed Ethnicity group

Among the patients who stopped MTX due to suspected toxicity, 11 were able to recommence MTX therapy, resulting in 33 (4.7%) discontinuing permanently. The 33 patients who were not rechallenged included 14 patients who had pulmonary symptoms due to ILD, nine with severe gastrointestinal intolerance opted not to recommence therapy, five patients had persistent haematological abnormalities (three had leucopaenia and two had thrombocytopaenia), and one had persistently elevated liver enzymes. Among the four patients who had an infection, three had persistent low disease activity and were not recommenced on MTX, while the attending physician chose not to recommence treatment in the fourth patient.

The 33 patients who stopped MTX comprised of 17 (4.0%) Africans and 16 (7.0%) Indians. There was no significant difference (p = 1.822) in MTX discontinuation due to intolerance or toxicity between the two groups.

The most common reasons for stopping MTX for factors other than adverse effects in 39 (5.6%) patients were iron deficiency anaemia in 24 (3.4%), desiring conception in seven, pregnancy in four, and a decline in renal function and malignancy in two patients each respectively. Fourteen of these patients were recommenced on MTX therapy – they included 10 patients who had iron deficiency anaemia, two patients who were pregnant and two patients who desired conception. Thus, overall, 25 patients (3.6%) discontinued MTX due to factors unrelated to toxicity and were not rechallenged with MTX. These 25 patients included 14 who had iron deficiency anaemia (seven had persistent severe anaemia, two were in remission, and five were lost to follow up), five who desired pregnancy (three of them were lost to follow up and two were still planning to conceive) and the two who were pregnant wished to breast feed and conceive again. The two patients with impaired renal function and one patient with malignancy, were not considered for rechallenge, and the second patient with malignancy was lost to follow up.

## Discussion

We report the reasons for MTX discontinuation in a multi-ethnic RA population in Durban, SA. The median duration of MTX therapy for all patients was 67 (39—106) months and was 68.5 (40.0—108.7) months in females which was significantly greater (p = 0.014) than 60.0 (30.0—84.0) months for males. The majority of our 695 patients were African Blacks (61.2%) and Indians (32.8%). Methotrexate was initially discontinued in 44 (6.3%) due to toxicity, but as 11 patients were successfully rechallenged, only 33 (4.7%) stopped treatment because of toxicity or intolerance. A further 39 (5.6%) stopped MTX for reasons other than toxicity, and as 14 of them were successfully rechallenged, 25 (3.6%) remained off treatment for factors unrelated to toxicity. In this relatively large study of 695 RA patients, we found a low MTX discontinuation rate of 8.3% due to toxicity, intolerance or social factors. We noted that none of our 15 patients of Mixed Ethnicity and 27 Whites discontinued MTX due to toxicity (one White patient discontinued as a result of pregnancy while the second patient who stopped as a result of iron deficiency anaemia was able to recommence therapy).

The reasons for discontinuation of MTX are many, and they include drug intolerance or toxicity. The most common adverse effects with MTX therapy are gastrointestinal intolerance, elevation of the liver enzymes, respiratory symptoms, haematological abnormalities, cutaneous abnormalities and possibly infections [14]. However, the routine use of folic acid has resulted in a reduction in the frequency of gastrointestinal side effects such as nausea, vomiting and abdominal pain, protection against elevation of serum transaminases, and an improvement in compliance because of a reduction in patients stopping their MTX therapy for these reasons [15]. Factors other than adverse effects which result in discontinuation of MTX are many and include lack of efficacy, and factors unrelated to drug effects such as a desire to conceive, pregnancy, remission, co-morbidities such as renal disease, poor adherence or patient choice [8, 14]. Kinder et al. noted that MTX was well tolerated in their 673 patients (81.9% with RA) and the probability of continuation after five years was 0.74 [14]. Curtis et al. conducted a systematic review of 29 studies and found that persistence (defined as the time from initiation to discontinuation) of MTX therapy ranged from 50—94% at one year to 25—79% at five years of therapy, and the main reason for withdrawal of therapy in 23—79% of patients was lack of tolerability [16].

In view of the wide variation in the tolerance of MTX in different studies, we compared the prevalence and reasons for discontinuation of MTX therapy in our patients with similar studies in other parts of the world as shown in Table 3 [2, 8, 14, 17–26]. Even though many patients experienced adverse effects, much smaller numbers had to discontinue MTX. We noted that there was considerable heterogeneity among these studies. Study differences were related to the characteristics of patients selected. Few studies had only RA patients while others included patients on MTX for other indications. The variable duration of therapy, and information about the use of folic acid was not available for all studies. van Ede noted that elevated alanine transaminase (ALT) values led to MTX discontinuation in only 11 (4.0%) of 274 patients on folic acid or folinic acid compared to 35 (25.5%) of 137 RA patients who did not receive folic acid [22]. Keysser et al. noted a very high rate of adverse effects and MTX withdrawal rate as folic acid was not used routinely in their patients [24].

**Table 3:** Comparison of the reasons for methotrexate discontinuation due to adverse effects and factors other than adverse effects

First author	Country/Year	Duration of MTX (months)	Number with RA	Adverse effect ^A^ n (%)						Reasons for discontinuation other than toxicity		Total stopped MTXn (%)
				GIT	Haemat-ological	Liver	Lung	Other	Stop MTX Total n (%)	Reasons for discontinuation n (%)	Stop MTX n (%)	
Present study	South Africa 2024	67.0 (39.0–106.0) ^B^	695	**9 (1.3)**	**5 (0.7)**	**1 (0.1)**	**14 (2.0)**	**4 (0.6)**	**33 (4.7)**	Pregnant 2 (0.3); planning pregnancy 5 (0.7), Severe anaemia 14 (2.0), fall in renal function 2 (0.3), malignancy 2 (0.3)	**25 (3.6)**	**58 (8.3)**
Mustafa [17]	Pakistan 2022	2.5 years	411	49 (11.9)	42 (10.2)	14 (3.4)	0	0	**17 (4.1)**		**9 (2.2)**	**26 (6.3)**
Oulkadi [18]	Morocco 2022	2.0 (0.5–27.8) ^C^ years	199	**40 (20.1)**	**4 (2.0)**	**18 (9.0)**	**2 (1.0)**	**3 (1.5)**	**66 (33.2**)	Ineffective 12 (6.0), other 10 (5.0)	**22 (11.1)**	**88 (44.2)**
Zongo [10]	Burkina Faso 2019	30.2	135	32 (25.9)	3 (2.2)	6 (4.4)	6 (4.4)	11 (8.1)	**8 (5.9)**	MTX shortage (10.4%), pregnancy related concerns (2.2%), financial (3.7%), other 5.1%	**29 (21.4)**	**37 (27.4)**
Nikiphorou [8]	UK 2014	10 (0.3–122.3) ^C^	762	**65 (8.5)**	**23 (3.0)**	**24 (3.2)**	**50 (6.6)**	**38 (5.0)**	**200** **(26.2)**	Ineffective 32 (12.4); not indicated 17(6.6), patient choice 14 (5.4) unknown in 14 (5.4)	**60 (7.9)**	**260 (34.1)**
Khabbazi[19]	Iran 2014	19.76 (1—144) ^C^	809 608 RA	**2 (0.3)**	0	**4 (0.5)**	0	**4 (0.5)**	**10 (1.2)**	Pregnancy planning 17 (2.1) Compliance 9 (1.1), Ineffective 5 (0.6), Remission 89 (11)	**120 (14.8**)	**130 (16.1)**
El-Zorkhany [20]	Egypt 2013	NR	111	**26 (23.4)**	**1 (0.9)**	**2 (1.8)**	**1 (0.9)**	**1 (0.9)**	**31 (27.9)**	Ineffective 1 (0.9), pregnancy planning 3 (2.7), poor compliance 3 (2.7) other 8 (7.2)	**15 (13.5)**	**46 (41.4)**
Malemba [21]	DRC 2013	20	98	0	0	**1(2.0)**	**1 (2.0)**	0	**2 (3.9)**^*E*^	Lost to follow up 36 (36.7), death -cause unknown 4 (4.1), < 20 months 7 (7.1) ^F^	**40 (40.8)**	**42 (42.8)**
Kinder [14]	UK 2005	NR	673 551 RA	20 (3.6)	29 (5.3)	29 (5.3)	18 (3.3)	47 (8.5)	206 (37.4)^*G*^	Ineffective or patient choice 72 (10.7)	**72 (10.7)**	**NR**
Van Ede [22]	Netherlands 2001	48 weeks	411	**20 (4.9)**	**3 (0.7)**	**46 (11.2)**	**4 (3.6)**	**24 (5.8)**	**97 (23.6)**			
Emery [23]	Multinational2000	52 weeks	498	168 (33.9)	0	82 (16.3)		149 30.0)	**74 (14.9)**	Ineffective 15 (3.0), lack of compliance 14 (2.8), death 5 (1.0), other 3 (0.6)	**37 (7.4)**	**111 (22.3)**
Keysser [24]	Germany 1999	4.6 years	371	145 (39.1)	69 (18.7)	44 (11.9)	13(3.4)	63 (17)	**59 (15.9)**	Ineffective 161 (43.4), other 4 (1.1)	**165 (44.5)**	**224 (60.4)**
Bologna [25]	France 1996	36.1 ± 28.0^H^ (*n* = 416)^I^	452	87 (19.2)	19 (4.2)	56 (12.4)	28 (6.2)	78 (17.3)	**63 (13.9)**	Ineffective 20 (4.4), extraarticular features 12 (2.7) and other in 8 (1.8), + 11 deaths	**40 (8.8)**	**114 (25.2)**
Alarcon [26]	USA 1989	27 ± 20.2^H^	152	25(16.4)*	9 (5.9)	0	3(2.0)	20 (13.2)	47(30.9)**	Surgery 6 (3.9); ineffective 6 (3.9), other 19 (12.5%	**31 (20.4)**	**78 (51.3)**
Salliot [2]	21 studies Review 2009	26.5 (27–132)^D^	3463	1065 (30.8)	179 (5.2)	640 (18.5)	84 (2.4)	500 (14.4)	**315/3007** **(10.5)**			

*RA* rheumatoid arthritis, *GIT* gastrointestinal, *NR* not reported

^A^ Prevalence of adverse effects is shown here; the bold figures indicate numbers and percent who discontinued MTX due to adverse effects.

^B^ Median and interquartile range.

^C^ Median (range).

^D^ The reasons for stopping other than toxicity are reported for all 809 patients.

^E^ Only 51 patients who received MTX for ≥ 20 months were analysed. ^F^ Seven patients who received < 20 months of MTX were not analysed. ^G^ Some of these RA patients were able to recommence MTX, actual number of RA patients are not reported. ^H^ Mean ± SD. ^I^ Mean duration of RA in the 416 patients who were < 65 years while the 53 patients who were ≥ 65 years had a mean duration of RA was 28.2 ± 25.5

^*^ Includes liver, ** Some patients had more than 1 adverse effect

The low prevalence of intolerance or toxicity of 4.7% in our study is similar to the prevalence of 1.2% and 4.1% reported from Iran and Pakistan respectively [17, 19]. It is considerably lower than the 26.2%, 30.9% and 33.2% reported from the UK, United States of America, and Morocco respectively [8, 18, 26]. Apart from intolerance or toxicity, Table 3 shows that other reasons for discontinuation of MTX were lack of efficacy, pregnancy or the desire to conceive, remission, compliance and patient choice. It is likely that socioeconomic factors contributed to 36 (36.7%) of the patients in the Democratic Republic of Congo (DRC) and 29 (21.4%) Burkina Faso being lost to follow up or being unable to access medical services or obtain regular medication [10, 21].

The most common adverse effects reported on MTX therapy in this study are gastrointestinal intolerance which includes nausea, abdominal pain and vomiting as shown in Table 3. Many patients are able continue MTX therapy despite these effects, while others may improve with a dose reduction, temporary discontinuation or regular use of folic acid therapy. Bologna et al. reported discontinuation of MTX due to toxicity in 63 (13.9%) of their 452 patients even though 87 (19.2%) had gastrointestinal adverse effects and 56 (12.4%) had elevated liver enzymes. A Dutch study of 291 patients (249 RA) on MTX noted that 123 patients (42.3%) experienced at least one gastrointestinal symptom, but only 32 (11%) patients discontinued MTX [27].

An increase in liver enzymes is common in RA patients on MTX but these are often mild and do not require discontinuation of treatment. A Swedish study of 213 RA patients noted elevated levels of ALT in 70% of their patients but only 7 (3.3%) patients had to discontinue MTX [28]. An analysis of the findings in 3808 RA patients treated for an average of 55.8 months in 27 prospective studies, showed that 769 (20.2%) had at least one episode of a raised liver enzyme, and only 3.7% stopped MTX because of liver toxicity [2]. Khabbazi et al. studied 809 patients (608 of whom had RA) on MTX therapy and found elevation of the liver enzymes in 108 (13.3%) patients. These patients required dose adjustment or temporary cessation, and only four patients (0.5%) required permanent discontinuation. In our study, only two patients (0.3%) discontinued MTX for hepatotoxicity and one of them continued treatment after a successful rechallenge. Table 3 shows that discontinuation of MTX due to liver injury was very low in Iran, Egypt and DRC, and was less than 5% in most of these studies.

The association of MTX with subacute hypersensitivity pneumonitis, a potentially lethal condition which usually occurs early in the course of therapy, is well established [29]. Earlier case series reported an increase in the risk of MTX pneumonitis in patients with pre-existing lung disease and therefore caution was advised with MTX use in patients with lung disease [30]. In 1997 Kremer et al. reported a series of 29 patients with MTX associated lung injury [30]. They also reviewed the clinical and radiographic findings in 86 patients with MTX induced pulmonary toxicity and noted that 72% of them had interstitial disease and 13% had interstitial and alveolar disease. Respiratory symptoms were associated with discontinuation of MTX in 6.6% and 6.2% of patients in a UK and French study respectively in Table 3 [8, 25]. Based on the earlier concerns about the possibility of MTX associated ILD, our 14 (2.0%) patients stopped MTX therapy when they developed new onset of respiratory symptoms and were found to have ILD [31].

A meta-analysis of 22 randomized controlled trials comprising 8584 patients, of whom 4544 were on MTX, showed a small but significant increase in all respiratory adverse events, and respiratory infections compared to comparator DMARDs [31]. Salliot et al. analyzed the pooled data from 21 prospective studies of 3463 patients and found MTX pneumonitis in only 15 of 3463 (0.43%) of patients, suggesting that non-infectious pulmonary manifestations are less common than reported in earlier studies [2]. A study of two large early RA inception cohorts’ of 2701 patients found that ILD occurred in 39 (2.5%) of 1578 MTX exposed patients and 53 of 1114 (4.8%) of patients not exposed to MTX. Based on their findings, the authors concluded that there was no association between incident RA-ILD and MTX therapy, and that MTX may have a protective role in delaying the onset of RA-ILD [32]. A recent retrospective international case control study of 410 patients with RA and ILD were compared to 673 RA patients with ILD [33]. The authors found that patients with RA on MTX were less likely to have ILD, and among patients with RA and ILD, the ILD was detected at a later stage [33]. Therefore, recent studies indicate that apart from MTX associated hypersensitivity pneumonitis, the presence of ILD is most likely due to RA than MTX.

There are conflicting reports whether MTX is associated with an increased risk of infection [34]. A literature review by Maclean et al. in 2009 noted that patients with RA have an increased risk of infection compared to controls, the risk being highest in patients with severe disease, concomitant use of corticosteroids and co-morbidities, and that low dose MTX was not associated with an increased risk of infection [35]. A systematic review by Baradat et al. noted that there was no increased risk of infection with the addition of MTX to biologic DMARD therapy compared to biologic DMARD therapy alone [36]. However, a meta-analysis of randomized controlled trials by Conway and Ibrahim found that MTX is associated with an increased risk of infection, especially respiratory infections [31, 34]. In our study, eight (1.2%) patients developed an infection, with four patients recommencing MTX after treatment of the infection. Thus only 4 (0.6%) patients discontinued MTX after an infection. We also noted that infection was not a major reason for discontinuation of MTX in the studies reviewed in Table 3.

The cytopaenias in patients on MTX may improve with dose reduction or temporary discontinuation of MTX. However, it is also important to consider the contribution of co-morbidities such as renal disease and concomitant drug therapy. There was a low prevalence of haematological abnormalities of around 5% or less in most of the studies (Table 3). We found that only 5 (0.7%) of our patients discontinued MTX due to haematological abnormalities. The low prevalence of cytopaenias is also noted in the studies from Iran, DRC, Egypt, Morocco and UK in Table 3 [8, 18–21].

Information about the relatively good safety profile and low prevalence of serious adverse effects with low dose MTX has come from the Cardiovascular Inflammation Reduction Trial (CIRT) which studied patients with previous myocardial infarction or multivessel coronary disease without infarction [37]. In the CIRT trial, MTX in a dose of 15 to 20 mg weekly did not result in a reduction of cardiovascular events in 2391 patients (median age 65.6 years) with stable atherosclerosis who did not have inflammatory arthritis compared to 2395 controls (median age 66 years) [37]. However, MTX was generally very well tolerated – even though patients on MTX had a significantly higher incidence of gastrointestinal, liver, pulmonary adverse effects and infections compared to placebo [38]. Although the incidence of raised ALT and aspartate transaminase (AST) three times the upper limit of normal, and leucopaenia were increased, the prevalence of these effects was relatively low compared to controls (ALT 3.68% vs 0.07%; AST 1.63% vs 0.88%, and leucopaenia in 10.07% vs 7.18%) [38]. Sparks et al. found that patients on low dose MTX had 13 severe pulmonary adverse effects (0.5%) and seven cases of possible pneumonitis (0.3%) vs eight (0.3%) and one (< 0.1%) in the placebo group, respectively [39]. They concluded that pulmonary adverse effects were uncommon but were more likely to occur in patients on low dose MTX.

### Strengths and limitations

The strength of this study is that it provides information about MTX intolerance and toxicity in a relatively large population of African Blacks in sub- Saharan Africa and found that there were no differences compared to Indians. All patients were managed in a single centre, received folic acid supplementation and had access to a constant supply of medication. As this was a retrospective study, all patients were not managed according to a standard protocol. Patients who experienced minor side effects or had mild elevations of liver transaminase which required short term discontinuation or dose reduction may not have been recorded. The disease activity scores were also not available in all patients.

## Conclusion

In conclusion, we report our single centre experience of a low prevalence of MTX intolerance or toxicity in a multi-ethnic population in Durban, SA. We also report the reasons for MTX discontinuation for factors other than adverse effects and compare our experience with findings in other parts of the world. We note that although gastrointestinal symptoms and liver enzyme abnormalities are common, with the use of folic acid, MTX discontinuation is necessary in only a small proportion of these patients. Although MTX pneumonitis is well documented as an association of MTX therapy, it is uncommon. Methotrexate, therefore can be used safely in sub-Saharan Africa as the initial drug of choice in RA.

## Data Availability

The data are available from the corresponding author on request.
